# Mechanical Sensors for Cardiovascular Monitoring: From Battery-Powered to Self-Powered

**DOI:** 10.3390/bios12080651

**Published:** 2022-08-17

**Authors:** Chuyu Tang, Zhirong Liu, Linlin Li

**Affiliations:** 1 School of Physical Science and Technology, Guangxi University, Nanning 530004, China; 2 Beijing Institute of Nanoenergy and Nanosystems, Chinese Academy of Sciences, Beijing 101400, China; 3 School of Nanoscience and Technology, University of Chinese Academy of Sciences, Beijing 100049, China

**Keywords:** mechanical sensors, cardiovascular disease, pulse wave, blood pressure, heart rhythm, endocardial pressure, cardiac output

## Abstract

Cardiovascular disease is one of the leading causes of death worldwide. Long-term and real-time monitoring of cardiovascular indicators is required to detect abnormalities and conduct early intervention in time. To this end, the development of flexible wearable/implantable sensors for real-time monitoring of various vital signs has aroused extensive interest among researchers. Among the different kinds of sensors, mechanical sensors can reflect the direct information of pressure fluctuations in the cardiovascular system with the advantages of high sensitivity and suitable flexibility. Herein, we first introduce the recent advances of four kinds of mechanical sensors for cardiovascular system monitoring, based on capacitive, piezoresistive, piezoelectric, and triboelectric principles. Then, the physio-mechanical mechanisms in the cardiovascular system and their monitoring are described, including pulse wave, blood pressure, heart rhythm, endocardial pressure, etc. Finally, we emphasize the importance of real-time physiological monitoring in the treatment of cardiovascular disease and discuss its challenges in clinical translation.

## 1. Introduction

Cardiovascular disease (CVD) and its complications are considered one of the leading worldwide causes of death [1]. It can also be referred to as a disease of the vascular system, which includes abnormal symptoms that arise from the blood supply to the heart, brain, and other vital organs [2,3]. Common CVDs include ischemic heart disease, stroke, and congestive heart failure [4]. They are all known for their high morbidity, disability, and mortality. Since CVD is mainly caused by hypertension, hyperglycemia, dyslipidemia, obesity, and lack of exercise, there has been a trend toward younger people in recent years [5]. Most cardiovascular-related mortality can be prevented through early monitoring followed by timely interventions, such as improved medication management and health care delivery [6]. The current treatment of CVD is a long-term process, and regular monitoring can significantly reduce the risk of CVD recurrence.

Clinically, CVD is usually assessed by electrocardiogram (ECG), Doppler ultrasound, arteriography, computer tomography (CT), etc., which can accurately diagnose the disease condition [7,8,9]. Moreover, some patients with arrhythmia and myocardial ischemia require real-time ECG monitoring [10]. However, traditional cardiovascular monitoring is often limited by the scarcity and inconvenience of medical resources and the application of bulky and expensive equipment for episodic diagnosis in hospital settings. In some less developed countries and in low-income populations, many people are at high risk of CVDs, and it is challenging to obtain a definitive diagnosis [1,7,11]. In the early 21st century, the use of sensors and mobile Internet began to provide a platform to continuously monitor vital signs, including respiration, pulse, heart rate, blood pressure, etc. [6,7]. The extensive material sources and diverse structural designs help to enhance the sensitivity and flexibility of the sensors and enable close attachment of the devices to non-planar tissue surfaces, which is essential for robust physiological measurements [12,13,14,15,16,17].

Both optical methods (i.e., photoplethysmography (PPG)) and mechanical methods (i.e., pressure/strain sensors) have been extensively investigated for the acquisition of physiological information from the cardiovascular system [7]. PPG methods measure blood volume changes during each cardiac cycle by irradiating tissue and calculating light absorption in the vascular system [18]. Although the PPG sensor is more mature and can be easily integrated into a variety of consumer electronics, it consumes relatively high electrical power, typically in the range of 10–100 mW for continuous monitoring. Its signal easily interferes with the surrounding environmental factors, such as skin color and sweat perturbation sensitivity [19,20]. Significantly, reducing optical power usually compromises signal quality, i.e., low signal-to-noise ratio, and therefore is not a viable solution [7]. These limitations present significant challenges in identifying detailed information about the physiological form carried by the pulse waveform signal. In contrast, mechanical methods can obtain information directly, reflecting the pressure waves in the vasculature by attaching a pressure or strain sensor to the surface of the vascular system or the skin close to the arteries [21,22]. Moreover, they are relatively more energy efficient and typically operated in the nanowatt to microwatt range. In addition, the electrical signal measured by mechanical sensors directly reflects the arterial pressure waveform, which provides additional information for diagnosing CVD. Thus, mechanical methods can provide a more accurate, compact, and energy-efficient solution for long-term CVD pressure monitoring.

This review presents recent advances in mechanical sensors applied to the cardiovascular system. First, four basic mechanical sensors and their working principles are described. Then, the physio-mechanical mechanisms of the cardiovascular system are elaborated, and different methods of physiological information monitoring are discussed. In addition, the application prospects from battery-powered to self-powered sensors are discussed from an energy perspective. Finally, the necessity to optimize the current practical monitoring of the cardiovascular system is summarized. It is intended to reduce the disjunction between the two fields of mechanical sensing and physiological signal acquisition to safeguard cardiovascular health.

## 2. Sensing Mechanism

The main flexible sensors for cardiovascular vital signs monitoring based on mechanical methods are strain sensors and pressure sensors, which can be monitored in a wearable or implantable form. Specifically, the basic sensing mechanisms of these sensors include capacitive [12,14,16,23,24,25], piezoresistive [26], piezoelectric [27,28,29,30], and triboelectric effects [31,32,33].

**Figure 1:**
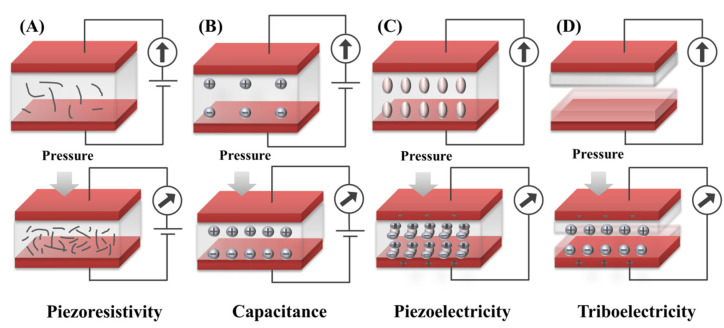
Schematic illustration of four sensing mechanisms: (**A**) Piezoresistivity; (**B**) Capacitance; (**C**) Piezoelectricity; (**D**) Triboelectricity.

The piezoresistive sensor is mainly based on the piezoresistive effect. The piezoresistive effect refers to the change of the resistance of pressure-sensitive materials under pressure excitation, which leads to the change of the output electrical signal. Another detection principle is based on the pressure-induced change in the geometrical morphology of conductive materials, which causes the change of contact resistance. Sensors manufactured based on the piezoresistive effect are driven by external forces that deform the dielectric material and indirectly change the distribution and contact state of the internal conductive filler, thus causing a change in the resistance of the dielectric material (Figure 1A).

Capacitive sensors convert the perceived mechanical load into a change of capacitance value in the device. The principle of capacitance for a typical parallel plate structure (Figure 1B) is defined as C=εS/d, where *S* is the area of the electrode plates facing each other, *d* is the distance between the two plates, and *ε* is the dielectric constant of the dielectric material. When a certain pressure is applied to the capacitor, the displacement of the electrode plates will be caused, generating a change in capacitance. Therefore, pressure sensing can be realized based on this principle.

Piezoelectric sensors convert mechanical forces into electrical signals based on piezoelectric materials and the piezoelectric effect. Piezoelectric materials are a class of dielectric materials with a non-centrosymmetric structure. When they are deformed by external stress or strain, a separation of positive and negative charges occurs within the material, producing an electrical potential difference between the upper and lower electrodes. The nature of the potential difference arises from the shift of the positive and negative charge centers in opposite directions, resulting in a micro-scale dipole moment in the unit cell (Figure 1C). Thus, the external pressure can be determined by measuring the potential difference.

Triboelectric sensors refer to a device that is capable of producing an electrical response to pressure stimuli based on the triboelectric effect and electrostatic induction. When two materials with different triboelectric properties are in contact with each other, there are equal and opposite charges on the surfaces of the two materials due to the triboelectric effect. The charge amount depends on the triboelectric properties difference between the two friction materials. The more the difference in triboelectric properties, the greater the amount of opposite charge on each surface, generating a potential difference. Driven by external pressure, the inductive charges are caused on the electrodes due to electrostatic induction. The charge flows between the two electrodes via the external circuit until an equilibrium is reached, producing an electrical output for sensing (Figure 1D).

Although different in mechanism, they are generally similar in structure, consisting of electrodes and active sensing components. For all of these sensors, the active sensing component changes in response to strain or pressure, resulting in a change in resistance, capacitance, or induced charge, which further changes the electrical signal output for sensing. Resistive and capacitive sensors require an external power source for sensing, whereas piezoelectric and triboelectric sensors can convert pressure or strain directly into an electrical signal for self-powered sensing [22]. Generally, macro-/micro-/nanostructural design on the surface of these sensing materials can increase the effective contact area and force perception ability, thereby improving the sensitivity of the sensor and enabling it to sensitively detect weak external pressure changes [12,14,23,24,25,34,35].

## 3. Biomechanical Monitoring in the Cardiovascular System

The cardiovascular system includes arteries, capillaries, and veins. Among all organs in the human body, the heart is relatively homogeneous in structure and function. By beating vigorously, the heart transmits enough blood to various organs in the human body to meet the metabolic needs of individual physiological functions. For this reason, the heart can be described as a unidirectional pump in series. From this characteristic, the heart must provide sufficient force to expel the blood it receives, respond to the body’s needs by varying the amount of blood expelled per unit time, and ensure unidirectional flow without reflux. As a biological pump, the heart is physiologically active. If any pump component is structurally or functionally compromised or malfunctions, it will result in diminished efficiency or, worst case, even complete cessation of physiological function. Furthermore, understanding each component of the heart as a pump helps to understand the underlying factors in CVD.

**Figure 2:**
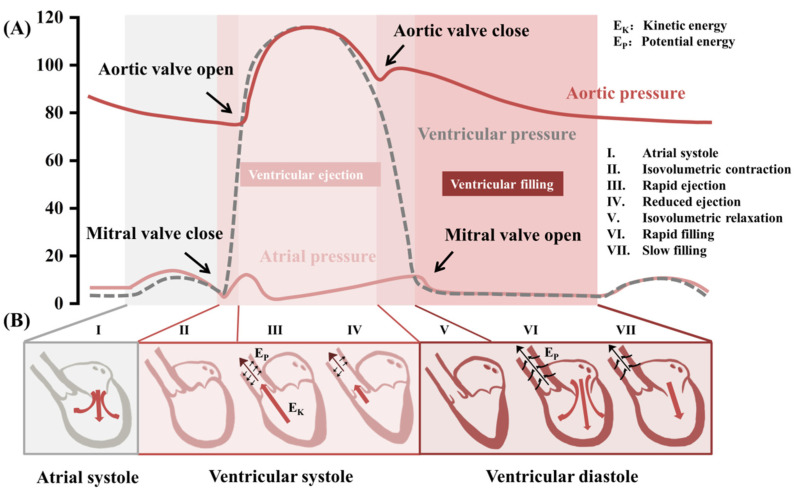
(**A**) The waveforms of aortic blood pressure versus ventricular and atrial blood pressure. (**B**) Cardiac cycle: the energy released by ventricular systole can be divided into two main components: the kinetic energy (E_K_) that drives the rapid flow of blood and the potential energy (E_P_) stored in the vascular wall.

A vascular wall is a hollow tube with a multi-layered composite structure that withstands blood pressure and is bound by extra-tubular tissues, usually consisting of inner, middle, and outer structures. The mechanical properties of the vessel mainly depend on the three components of the middle layer: elastic fibers, collagen fibers, and smooth muscle. Vascular wall compliance indicates one of the important clinical indicators of the arterial system’s cushioning function in terms of elasticity or stiffness [36]. The closer to the heart, the higher the percentage of elastic fibers in the arterial vessel wall, and thus the more elastic it is. By contrast, arteries farther from the heart are less compliant. With the intermittent contraction and diastole of the heart, the blood flows throughout the arterial vasculature in the form of pulse waves [37]. It is usually sensed in the form of pressure waves. A typical pulse wave consists of two parts: ascending and descending branches. The ascending branch indicates the sudden expansion of the artery during ventricular systole, while the descending branch indicates ventricular diastole, as shown in Figure 2A.

### 3.1. Pulse Wave

The mechanical pressure sensor has high sensitivity and fast response time, which is more realistic in detecting the pressure signal generated by the pulse wave and can more truly obtain the pulse wave signal. Arteries are tubes composed of elastic connective tissue and muscle. Due to the alternating contraction and relaxation of the ventricle during the cardiac cycles, the pulse periodically expands and returns, producing a pulse signal. Usually, the pulse also corresponds to the cardiac cycle frequency and is expressed in beats per minute (b.p.m.). In existing wearable healthcare systems, two types of pulse pressure sensors are commonly used [22]. One is a battery-powered pulse sensor, including piezoresistive, capacitive, and optical sensors that require an external power source to work. The other is self-powered pulse sensors, mainly piezoelectric and triboelectric pressure sensors, which can convert pulse waves into electrical signals.

**Figure 3:**
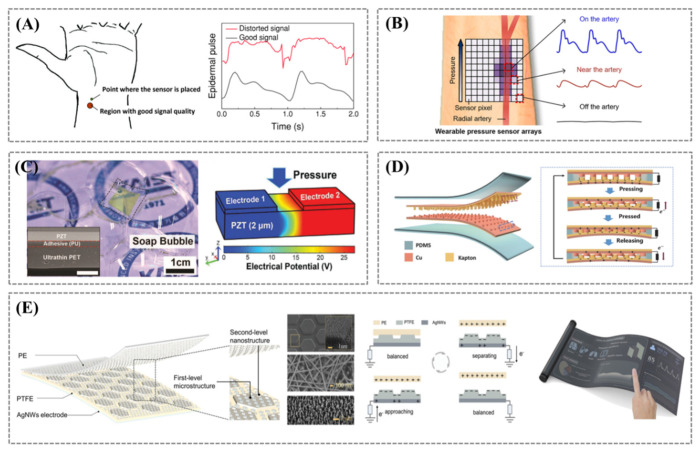
(**A**) Comparison of normal and distorted signals due to sensor misalignment in epidermal pulse measurement. Reprinted/adapted with permission from Ref. [38]. (**B**) Sensor arrays for locating arteries and acquiring accurate pulse waveforms. Reprinted/adapted with permission from Ref. [39]. (**C**) Ultrathin flexible PZT pressure sensor and the piezoelectric potential distribution, the inset indicates a cross-sectional SEM image (scale bar, 5 µm). Reprinted/adapted with permission from Ref. [40]. (**D**) Schematic diagram of self-powered ultrasensitive pulse sensor (SUPS) and its working principle. Reprinted/adapted with permission from Ref. [41]. (**E**) Schematic illustration of the ultrathin and flexible sensor (UFS) and its application for finger-touching measurement in single-electrode mode. Reprinted/adapted with permission from Ref. [42].

Measurement of the epidermal pulse by the traditional single-point mechanical sensor largely depends on the sensor’s precise placement. High-quality signals can be obtained only when the sensor is placed on the invisible arterial line. Misalignment of sensors with the peripheral artery (inappropriate wrist posture, such as drawing or folding skin pose) often leads to signal distortion and difficulty in information extraction, quickly reducing the signal amplitude and waveform fidelity (Figure 3A). Previously reported wearable sensors must be tightly fixed on the wrist while monitoring, for example, by adhesive tape to prevent position drift and dislocation [14,27]. At the same time, subjects are required to sit still and maintain optimal posture and wrist position during the measurement. To solve these problems, Fan et al. proposed a wearable liquid capsule pressure sensor (LCPS) for continuous and accurate monitoring of heart rate (HR) and blood pressure (BP) [38]. The liquid capsule consists of a flexible membrane and a filling fluid. By transmitting the pulsation signal detected by the capsule to an embedded piezoresistive sensor according to Pascal’s principle, the alignment accuracy between the pressure sensor system and the artery was incredibly relaxed to approximately 8.5 mm. For the 11 subjects (i.e., those wearing the LCPS without position calibration), the mean correlation coefficient between LCPS-derived and ECG-derived heart rate is 0.9944. The ECG was combined with the PPG and LCPS, respectively, to calculate the subject’s systolic and diastolic beat-to-beat BP values, and these data were compared with blood pressure values measured by cuff devices. The results demonstrate that the LCPS/ECG pair is as accurate as the typical PPG/ECG pair. This spatially insensitive, low-power (≈35 nW) LCPS can be easily installed by the user while ensuring the sensing ability of the sensor’s high-fidelity epidermal pulse signal. In addition, using the wearable pressure sensor array to measure the temporal and spatial pulse waves to obtain the best pulse waveform can also be regarded as a solution [18,39]. Baek et al. used inkjet printing technology to fabricate thin-film transistor (TFT) arrays integrated with thin sheets of highly sensitive piezoresistive sensors (Figure 3B) [39]. The operating voltage of TFT was strategically modulated to maximize pressure sensitivity (16.8 kPa^−1^) with low power consumption (101 nW). This wearable ultrathin pressure sensor array with 100 pressure sensing pixels created a two-dimensional distribution of pulse waves on the wrist. It can accurately measure the pressure signal and position of the artery to create a space-time pulse wave map that shows the position dependence of pulse amplitude. Thus, locating arterial lines can accurately extract the augmentation index, which is a parameter for evaluating arterial stiffness.

Taking advantage of the electronic skin tactile sensors, Sihong Wang’s research group developed a pressure sensor with high flexibility and sensitivity [35]. This sensor provided constant sensing performance under stretching. Through the cooperative design scheme of ionic capacitance sensing mechanism and mechanical layered micropyramid mechanism, a 98% strain insensitivity under 50% tensile strain was achieved, and the lower limit of pressure monitoring was 0.72 Pa. Remote physical diagnosis and treatment of patients can be achieved by using these flexible pressure sensors on pneumatically actuated soft manipulators. Quantitative pressure sensing on deformable surfaces and high precision tactile monitoring on stretchable surfaces will undoubtedly facilitate the future development of accurate detection of human or soft robot skin interactions.

In order to realize conformal contact between sensor and skin, Park et al. developed an ultrathin conformal piezoelectric sensor using Pb[Zr_x_,Ti_1−x_]O_3_ (PZT) [40]. High-quality PZT films were coated onto a sapphire substrate and annealed, and then the PZT films were peeled off and transferred onto an ultrathin polyethylene terephthalate (PET) substrate (4.8 μm). Gold forked finger electrodes were then assembled on the surface of the PZT film, which was so thin that the device could float on a soap bubble (Figure 3C). The piezoelectric sensor was attached to the human wrist by a bandage to monitor the pulse signal in real time and transmit it to a smartphone for pulse monitoring via a wireless transmission system.

Due to the tiny signal obtained by piezoelectric pulse sensor, a signal amplification system is often required to convert it into an electrical signal that can be analyzed, which puts forward a new demand for power consumption of wearable devices. Thus, highly sensitive and self-powered sensing based on fully active sensing technology offers a new idea for the detection of physiological pulse signals. Ouyang et al. used nanostructured metallic copper and polymer film as the friction layer and flexible polydimethylsiloxane (PDMS) as the encapsulation layer to produce a flexible self-powered ultrasensitive pulse sensor (SUPS) (Figure 3D) [41]. The output electrical signal of the self-activating, ultrasensitive pulse sensor can be transmitted by Bluetooth without signal amplification, providing early warning and diagnosis of cardiovascular disease. Wang et al. developed an ultrathin and flexible sensor (UFS) consisting of a polytetrafluoroethylene (PTFE), a polyethylene (PE) friction layer, and an AgNWs electrode layer [42]. The PTFE surface was modified with a multilayer microstructure, with a hexagonal microstructure at the first level and nanowires at the second level (Figure 3E). The results showed that the multilayer microstructured PTFE had an output voltage 7 and 2.7 times higher than the non-microstructured and single nanowire microstructured PTFE, respectively, with a sensitivity of 7.2 V kPa^−1^ and a response time of less than 4 ms. In addition, the sensitivity was up to 0.15 mV Pa^−1^ even at a static force of 1500 mN. Given these excellent properties, the UFS can capture fingertip pulse waves without constraint. More importantly, this work has successfully enabled a proof-of-concept for a healthcare system by integrating the UFS into mobile devices.

### 3.2. Blood Pressure

Blood pressure is the lateral pressure of blood against the wall of the blood vessel per unit area. Blood pressure is mainly determined by three factors: the volume of blood ejected from the heart into the arteries, the elasticity or stiffness of the arterial walls, and the rate at which blood flows out of the arteries [37]. During the cardiac cycle (Figure 2B), systolic blood pressure occurs when blood is ejected from the heart into the arteries. At the same time, diastolic blood pressure occurs when the heart rests between heartbeats. Usually, blood pressure is higher in the morning (or during wakefulness) and lower at night (or during sleep). The difference in blood pressure measured during these two periods may be associated with the incidence of several cardiovascular events, such as stroke, heart failure, and end-stage kidney disease [19,43,44].

Continuous blood pressure differs from single and ambulatory blood pressure. It refers to real-time arterial blood pressure changes whose waveforms and trends are clinically meaningful, which can be used for pacemaker optimization, automatic diagnosis of heart failure, haemodynamic assessment, and assessment of sleep dysfunction [43,44]. When arterial pressure varies during the cardiac cycle, the shape of the different signals associated with blood pressure also changes, as shown in Figure 2A [20]. In this section, we will discuss blood pressure measurement technology based on mechanical pressure sensing technology, its conversion mechanism, and the sensors used to enable accurate real-time monitoring.

#### 3.2.1. Wearable Blood Pressure Monitoring

**Figure 4:**
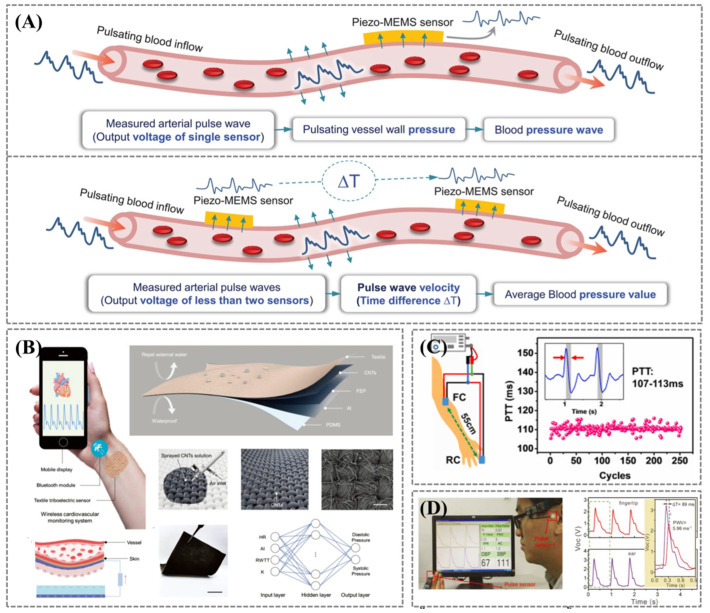
(**A**) The pressure transmission mechanism. Upper (PWA): The correlation between blood pressure waveform and arterial pulse piezoelectric response. Down (PTT): Time difference between two arterial pulse sensors to obtain blood pressure. Reprinted/adapted with permission from Ref. [46]. (**B**) Diagram illustrating a self-powered textile-based triboelectric sensor. The collected signal can be transmitted wirelessly to mobile phones, and the architecture of the supervised feedforward neural network is used for blood pressure prediction. Reprinted/adapted with permission from Ref. [47]. (**C**) Brachial–fingertip PTT and PWV monitoring via the self-powered ultrasensitive pulse sensors (SUPSs). Reprinted/adapted with permission from Ref. [48]. (**D**) A sensor system simultaneously monitors the pulse waves from the human fingertips and ears to read the participants’ PWV and BP values in real time. Reprinted/adapted with permission from Ref. [49].

Blood pressure is closely related to the hemodynamics of the cardiovascular system. As blood in the elastic ducts (arteries) is approximated as an incompressible fluid, energy transfer is mainly conducted through the vessel walls [37]. Vasoconstriction and diastole affect not only the blood volume but also the pressure pulsations within the vessels. Clinical blood pressure measurements are typically performed using auscultatory and oscillometric methods. Both of which require an inflatable cuff connected to an air pump and a gas pressure transducer to measure absolute air pressure values [19,20]. This principle of obtaining an average blood pressure value by measuring brachial blood pressure with a cuff is based on the assumption that brachial blood pressure accurately reflects central aortic pressure (CAP) [37]. Although simple and convenient, it cannot be measured continuously. To date, the most accurate way to measure blood pressure is to insert a pressure transducer directly into the aorta to detect the blood pressure [43], which enables continuous blood pressure measurement. However, this method is invasive and not suitable for long-term monitoring. The mechanical sensor-based cuffless method for continuous blood pressure offers the patient greater freedom of movement [19]. There are two common measurement methods: pulse pressure wave analysis (PWA) and pulse transit time (PTT), as shown in Figure 4A.

For PWA, the morphological characteristics of pulse waves can be considered as an objective description of the cardiovascular activity state when conducted in artery vessels and contains abundant physiological information in the human body system [25]. PWA assists in modeling the relationship between pulse waveform and blood pressure by combining the effects of hydrodynamics and vascular elasticity [45]. The research of blood pressure prediction based on PWA is mainly from two directions of parameter selection and model optimization.

Fang et al. showed a low-cost, lightweight, and mechanically durable textile triboelectric sensor that can convert subtle skin deformations caused by arterial pulsation into electrical energy for high-fidelity and continuous pulse waveform monitoring in mobile and sweaty environments (Figure 4B) [47]. Aided by machine learning algorithms, the textile triboelectric sensor can continuously and accurately measure systolic and diastolic blood pressure, and its accuracy was verified by the hospital’s commercial blood pressure cuff. The system supports one-click sharing of health data and data-driven cardiovascular diagnostics. Yi et al. developed a wireless wearable continuous blood pressure monitoring system [46]. Through kinetic theory, simulation, and experimental analysis, three kinds of correlations between piezoelectric response and blood pressure were revealed: integration, transition correction, and direct correlation. The feasibility of wearable continuous blood pressure monitoring without motion artifacts using a single piezoelectric sensor was verified (Figure 4A). These findings resolve the controversy over the piezoelectric response of pulse waves in the artery and provide better convenience than traditional systems based on pulse wave velocities between multiple sensors. It is expected to be employed to develop a portable and wearable continuous blood pressure monitoring device for early prevention and daily control of hypertension.

For PTT, it is possible to calculate BP values from the pulse wave by combining the statistic method with experimental measurements to estimate the coefficients associated with the linear model [38,50]. PTT is the time delay of pressure wave transmission between two parts of the artery (Figure 4A). Two PW signals are obtained from these two parts, and then a linear regression model can be established using PTT to measure the BP value. Therefore, accurate measurement of PTT in the experiment is the basis for monitoring cardiovascular indicators such as pulse wave velocity (PWV) and BP. At present, PTT can be obtained by a dual signal method:

$$\mathrm{PWV}=\sqrt{\frac{Eh}{\rho d}}=\frac{L}{\mathrm{PTT}}$$

$$\mathrm{BP}=\alpha \cdot {\mathrm{PWV}}^{2}+\beta$$

in which *α* and *β* are determined by the material properties and geometry of the artery. PWV represents pulse wave velocity; *E* represents arterial elasticity coefficient; *h* represents vascular thickness; *d* represents intra-arterial diameter; and *ρ* represents blood density. PTT is the time for the pulse wave to travel along the artery from one part of the artery to the distal part, and *L* is the distance between two points. The square of PWV is proportional to arterial elasticity coefficient *E* and inversely proportional to PTT. Developing new technology for dynamic blood pressure monitoring based on PTT or PWA needs the support of specific algorithms or mathematical models. Accurately obtaining the PWV or BP is a prerequisite and requires a calibration estimate with a medical sphygmomanometer.

Xu et al. presented a non-invasive multi-indicator cardiovascular monitoring strategy based on self-powered ultrasensitive pulse sensors (SUPSs) distributed over different arterial pulse locations [48]. The arm-fingertip PTT was obtained by extracting the difference in the peak position of the pulse waveforms sensed by the SUPSs (Figure 4C). Linear fitting with the average blood pressure measured by an electronic cuff sphygmomanometer, various cardiovascular indicators were monitored, including heart rate, pulse wave velocity, and blood pressure, which were highly consistent with those measured by commercial medical instruments. Meng et al. reported a flexible weaving self-powered pressure sensor (WCSPS) [49]. Surface nanowires were introduced into PTFE by plasma etching, enabling the single-electrode working mode of the WCSPS with ultra-sensitivity of 45.7 mV Pa^−1^ and ultra-fast response time of less than 5 ms. It was applied to actual measurements of 100 people aged 24 to 82 with varying health conditions. PTT was obtained by the delay between the fingertip signal’s peak point and the ear signal’s peak point, and an improved genetic algorithm calculated the BP value in real time (Figure 4D). The BP values measured by WCSPS compared to those measured by a commercial cuff-based device differed approximately 0.87% to 3.65%. They all demonstrate an efficient and economic real-time cardiovascular monitoring system.

#### 3.2.2. Implantable Blood Pressure Monitoring

Revascularization refers to restoring blood perfusion in narrow, occluded, or non-functional arteries through drugs or surgery to restore blood supply to corresponding ischemic organs [51]. Among them, stent implantation and vascular replacement are commonly used in clinical surgery [52]. Millions of cardiovascular stents are implanted annually in coronary artery disease [53]. However, intra-arterial stents may cause excessive growth of arterial tissue, leading to re-stenosis of the stent. In addition, some cancer or trauma patients require microvascular tissue transplantation [54]. After each operation, it is vital to ensure fluent blood flow to the new anastomosis.

Bao et al. reported a biodegradable sensor for wireless battery-free monitoring of arterial blood flow [55]. The prepared pressure sensor can be easily wrapped around arteries of all sizes due to its lightweight and flexible properties. Based on fringe field capacitance technology, it can measure arterial blood flow in both contact and non-contact modes. The sensor has the advantages of minimal latency, fast response time, excellent cycle stability, high robustness, and easy installation without disassembly, thus reducing the risk of vascular injury. Herbert et al. developed a vascular electronic system consisting of a wireless stent system, which integrated soft sensors to meet implantation and manipulation requirements [56]. A structured dielectric layer was fabricated using the aerosol jet printing method to improve the sensitivity and response speed of the capacitive sensor, and two stretchable interconnect pressure sensors were installed on the stent to monitor flow velocity changes in the artery. The system can monitor hemodynamics, including pressure, pulse rate, and flow, in real time and work at a communication distance greater than existing vascular sensors through inductive coupling, enabling wireless data transmission.

**Figure 5:**
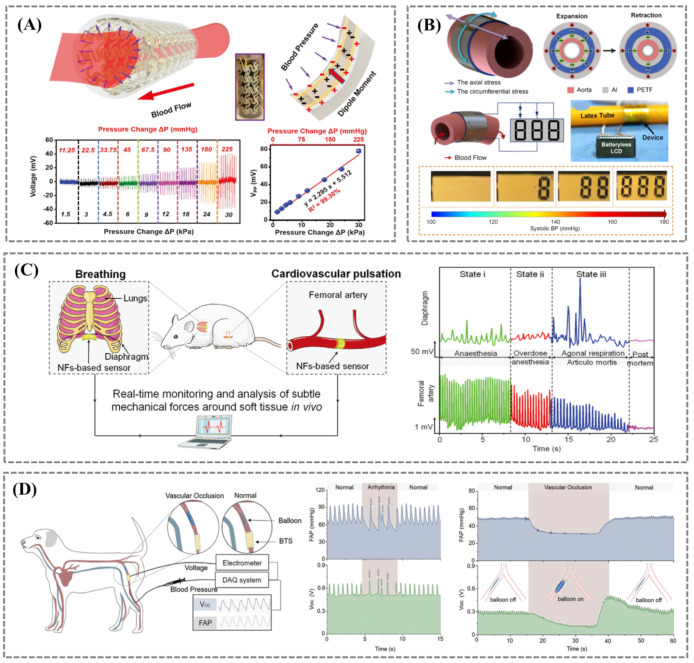
(**A**) Schematic showing the response of the piezoelectric effect to blood pressure in artificial artery. Reprinted/adapted with permission from Ref. [57]. (**B**) An implantable, self-powered, and visualized BP monitoring system. Diagram illustrating the circumferential stress in the expanding aortic wall, inducing the charge distribution of the piezoelectric sensor. Reprinted/adapted with permission from Ref. [61]. (**C**) Diagram of implantable PVDF/DA NF−based sensor for monitoring subtle mechanical pressure changes in vivo. Reprinted/adapted with permission from Ref. [63]. (**D**) Schematic of the bioabsorbable triboelectric sensor (BTS) monitoring physiological signals in vivo. Reprinted/adapted with permission from Ref. [64].

Self-powered capability is of great significance for future intelligent implants [57,58,59,60], and battery-free sensing of physiological signals in artificial blood vessels may be an ideal application. Li et al. developed an electric field-assisted 3D printing technique for fabricating in situ polarized ferroelectric artificial arteries (Figure 5A), providing battery-free real-time blood pressure sensing and occlusion monitoring capabilities [57]. The synergistic effect of potassium sodium niobate particle (KNN) and polyvinylidene fluoride (PVDF) polymer matrix produced excellent piezoelectric properties (d33 > 12 pC N^−1^). The ferroelectric material designed with a sinusoidal structure and 3D printing made the mechanical modulus close to the vascular level, allowing sensitive sensing of pressure changes (0.306 mV mmHg^−1^, R^2^ > 0.99) in the human blood pressure range (11.25–225.00 mmHg). Pressure sensors with high sensitivity can detect subtle changes in vascular motion, enabling early detection of partial occlusions (such as thrombosis) and preventing graft failure. Cheng et al. fabricated a self-powered implantable blood pressure sensor based on a piezoelectric thin film (PETF) (Figure 5B) [61]. The sensor’s output voltage had a suitable linear correlation to the systolic blood pressure of the aorta (R^2^ = 0.971) with a sensitivity of 14.32 mV mmHg^−1^. The implantation of the PETF into adult Yorkshire pigs allowed real-time monitoring and visual warning of hypertensive status without the need for an external power source. The fabrication of high-performance piezoelectric biomaterials is essential for the development of wearable and implantable biomedical devices. In 2019, Feng’s group reported the first miniature thrombus detector [62]. By preparing core/shell polyvinylidene fluoride (PVDF)/hydroxylamine hydrochloride (HHE) organic piezoelectric nanofibers (OPNs), the piezoelectricity, fatigue resistance, stability, and biocompatibility of the material were significantly improved. Then, the PVDF/HHE OPNs soft sensor was developed for monitoring tiny pressure changes in vivo. It exhibited ultra-high detection sensitivity and accuracy when implanted in pigs and can capture micro-pressure changes outside the cardiovascular wall. The output piezoelectric signal can reflect and distinguish the changes in cardiovascular elasticity, the onset of atrioventricular heart block, and thrombus formation in real time and synchronously. This biological information can be used for the early assessment and diagnosis of thrombosis and atherosclerosis, especially for postoperative recurrence of deep thrombosis. In the following year, they reported a one-step method to prepare core/shell PVDF/dopamine (DA) nanofibers (NFs) with high beta-phase content and self-aligned polarization properties [63]. The self-assembled core/shell structure was considered to be the key to the formation and alignment of the β-phase PVDF, where the solid intermolecular interaction between the -NH_2_ group on DA and the -CF_2_ group on PVDF enhances the alignment of polymer chains and promotes β-phase nucleation. The obtained PVDF/DA NFs exhibit significantly enhanced piezoelectric properties, excellent stability, and biocompatibility. Based on this, an all-fiber soft sensor was developed, which had high sensitivity and accuracy in detecting weak physiological mechanical forces generated by diaphragm movement and blood pulsation (Figure 5C). Thus, it can be employed to assess and prevent cardiovascular and respiratory diseases early.

Coincidentally, another class of self-powered stent concepts based on triboelectric nanogenerator (TENG) has been proposed. Kaveh Barri et al. proposed a self-sensing, biocompatible, and non-toxic stent fabricated from composite mechanical metamaterials, which can be self-powered by harvesting energy from arterial pulsations in blood vessels [58]. The stent can be deployed using a commercial balloon dilator catheter to continuously monitor local hemodynamic changes for tissue overgrowth and arterial restenosis. The proposed design is a proof-of-concept and does not optimize for optimal electrical and mechanical properties. However, incorporating the concept of self-powering into the design of implantable stents allows continuous measurement of applied forces and energy harvesting from external mechanical excitation. It is essential for designing advanced medical stents, demonstrating a promising strategy for incorporating versatility into intelligent medical systems for artificial biological systems.

Bioabsorbable electronic devices can be absorbed and become part of living organisms after their service life, thereby avoiding invasive secondary surgery, a new trend in the development of implantable sensors. In 2021, Ouyang et al. reported a pressure sensor based on the triboelectric effect between bioabsorbable materials, which can directly convert environmental pressure changes into electrical signals (Figure 5D) [64]. This bioabsorbable triboelectric sensor (BTS) had excellent sensitivity (11 mV mmHg^−1^), linearity (R^2^ = 0.993) and suitable durability (450,000 cycles). After implantation in large animals, it has a service life of 5 days with high efficiency (5.95%) and can successfully identify abnormal vascular occlusion events.

### 3.3. Heart Rhythm and Endocardial Pressure

Cardiovascular implanted electronic devices (CIEDs), such as pacemakers, cardiac resynchronization therapy (CRT) devices, and defibrillators, play key roles in reducing morbidity/mortality by monitoring, restoring, and regulating heart function [3,8,65]. A key indicator for diagnosing the pumping capacity of the heart is endocardial pressure (EP), such as atrial and ventricular pressure [10]. A piezoresistive pressure sensor for intracardiac catheters is the only effective way to obtain EP data clinically, but it is invasive to use and cannot collect data continuously for a long time [8,10]. In addition, the bulky external recorder attached to the cardiac catheter also brings a series of disadvantages, such as complicated operations and poor patient compliance [66]. In clinical practice, intermittent EP measurement may neglect transient/silent symptoms and lead to misdiagnosis of patients [67].

**Figure 6:**
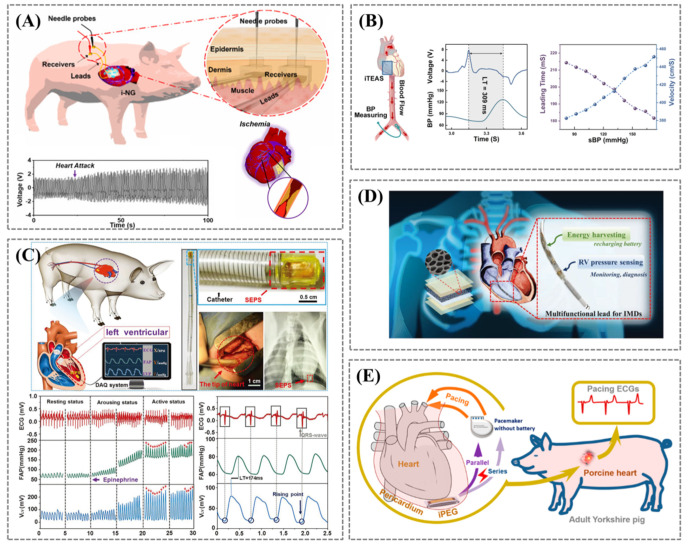
(**A**) Schematic of the i−NG system placed in pig, showing the voltage output during normal heartbeat and heart attack by blocking the coronary arteries causing ischemia. Reprinted/adapted with permission from Ref. [68]. (**B**) Schematic diagram of the mechanism for monitoring blood flow velocity by iTEAS. The red arrows indicate the direction of blood flow. Reprinted/adapted with permission from Ref. [69]. (**C**) The comparison of working signals from ECG, FAP, and SEPS in different states and the SEPS implanted into an Adult Yorkshire swine’s heart via minimally invasive surgery. Reprinted/adapted with permission from Ref. [67]. (**D**) Diagram showing a multifunctional pacemaker lead that combines energy harvesting and sensing strategies. Reprinted/adapted with permission from Ref. [70]. (**E**) Schematic illustration of the implantable piezoelectric generator (iPEG). Reprinted/adapted with permission from Ref. [71].

Self-powered sensors with low or zero power consumption and high performance for monitoring physiological signals (e.g., for active endocardial monitoring) will make strengths in real-time medical monitoring. Li et al. evaluated the safety of a soft and flexible implantable nanogenerators (i-NG) system for long-term in vivo cardiac implantation [68]. The system consisted of piezoelectric PVDF-based i-NG, leads, and receivers were implanted into the epicardial membrane of pigs for two months. As the heart expands during diastole, the PVDF membrane bends outward and generates an electrical potential. By temporarily occluding the left anterior descending branch (LAD) and inducing ischemia to mimic a heart attack, the i-NG responded immediately to ischemia with an increase in peak-to-peak voltage (V_pp_) from ~2.3 V to ~4.5 V (Figure 6A). Ma et al. present a self-powered, flexible, one-stop implantable triboelectric active sensor (iTEAS) for continuous monitoring of multiple physiological and pathological signs (Figure 6B) [69]. After the sensor is properly encapsulated and installed on the surface of the heart, the triboelectric layer separates and contacts with the systole and diastole of the heart and continuously outputs electrical signals through the coupling of contact electrification and electrostatic induction. The monitoring function remained excellent after 72 h of chest closure. The device can monitor heart rate with an accuracy of 99%. Atrial fibrillation, premature ventricular beats, and other arrhythmias can be detected in real time. The device had suitable biocompatibility after implantation for two weeks, proving its suitability for practical use. As a multifunctional biomedical monitor that requires no external power supply, the proposed iTEAS has great potential in the healthcare field.

Minimally invasive monitoring of changes in endocardial pressure (EP) has important clinical significance for heart failure patients with impaired cardiac function. Liu et al. proposed a miniaturized, flexible, ultrasensitive, and self-powered endocardial pressure sensor (SEPS) based on the triboelectric principle for real-time monitoring of EP [67]. The SEPS has an identical trend to the pressure signals obtained with commercial probes in the simulated fluid pressure environment in vitro. To verify the monitoring performance of the SEPS in vivo, minimally invasive catheters were implanted into the left atrium of male adult Yorkshire pigs. The arterial pressure catheter was placed in the left femoral artery to measure the femoral artery blood pressure (FAP). The voltage output of the SEPS during cardiac systole and diastole was collected separately and was almost perfectly synchronized with the FAP measured during adrenaline-controlled systole (Figure 6C). In vitro test results showed that the device oscillated more than 100 million times in a humid environment and had mechanical and electrical stability. In addition, the encapsulation layer had suitable haemocompatibility and a sufficiently low risk of causing hemolysis and activating clotting cascades. These advances contribute to the safe sensing of pressure conditions, diagnosis, and monitoring of cardiovascular diseases and have promising applications in the field of implantable health monitoring.

Self-powered implantable medical electronics that combine monitoring and disease treatment are part of an emerging medical revolution. Dong et al. proposed a strategy that combined cardiac energy harvesting and endocardial pressure sensing (Figure 6D) [70]. Energy-harvesting devices fabricated using advanced piezoelectric porous thin film can convert the mechanical motion generated by cardiac systole and diastole into electrical energy. On the one hand, right ventricular pressure changes can be monitored in real time to alert arrhythmias. On the other hand, it can be integrated with existing pacemakers or implantable cardioverter defibrillators (ICD) leads for cardiac pacing, thereby reducing the reliance on batteries and extending the life of pacemaker batteries by 20%. Li et al. reported a comprehensive strategy for directly powering a fully functional pacemaker that can pace pig hearts in vivo by harvesting the natural energy of the heartbeat without using any external energy storage components (Figure 6E) [71]. The generator included an elastic skeleton and two piezoelectric composites that can generate high output currents of 15 μA in vivo. This research is a promising step forward in the manufacture of self-powered pacemakers and the use of piezoelectric harvesting technology to solve the power supply problem for implantable medical devices. Ouyang et al. also demonstrated a fully implantable symbiotic pacemaker based on implantable TENG, which enabled energy harvesting and storage as well as cardiac pacing on a large animal scale [72]. The open circuit voltage of the implantable TENG can reach up to 65.2 V. Approximately 0.495 μJ of energy was obtained during every cardiac cycle, higher than the threshold energy required for endocardial pacing (0.377 μJ). The symbiotic pacemaker successfully corrected sinus arrhythmias and prevented deterioration. TENGs for implantable medical devices have the advantages of excellent output performance, high power density, and suitable durability and are expected to be applied in the therapeutic and diagnostic fields such as symbiotic bioelectronics in vivo.

### 3.4. Cardiac Output

Cardiac output (CO), the volume of blood ejected per minute from one ventricle, is one of the main determinants of the systemic oxygen supply and is a central parameter in the monitoring of haemodynamically unstable patients in intensive care and perioperative treatment. These include the invasive intermittent pulmonary thermodilution method using a pulmonary artery catheter (the clinical gold standard), the minimally invasive and non-invasive ultrasound Doppler method, bioimpedance method, and pulse wave analysis. Cardiac output (CO), the volume of blood ejected per minute from one ventricle, is one of the main determinants of the systemic oxygen supply and is a central parameter in the monitoring of haemodynamically unstable patients in intensive care and perioperative treatment [73,74]. The common methods for monitoring cardiac output include the invasive intermittent pulmonary thermodilution using a pulmonary artery catheter, the minimally invasive and non-invasive ultrasound Doppler and pulse wave analysis [75,76].

The pulmonary thermodilution method of CO monitoring enables a comprehensive assessment of cardiovascular function and is considered the clinical gold standard. However, it is rarely suitable for low- or moderate-risk non-cardiac surgery patients. The ultrasound Doppler technology, a well-established method of clinical haemodynamic measurement, transthoracic echocardiography (TTE), has emerged as a commonly used monitor in the cardiac operating room and ICU. However, there are some limitations, such as semi-continuous real-time monitoring, operator dependence, and the need for an accurate ultrasound window.

**Figure 7:**
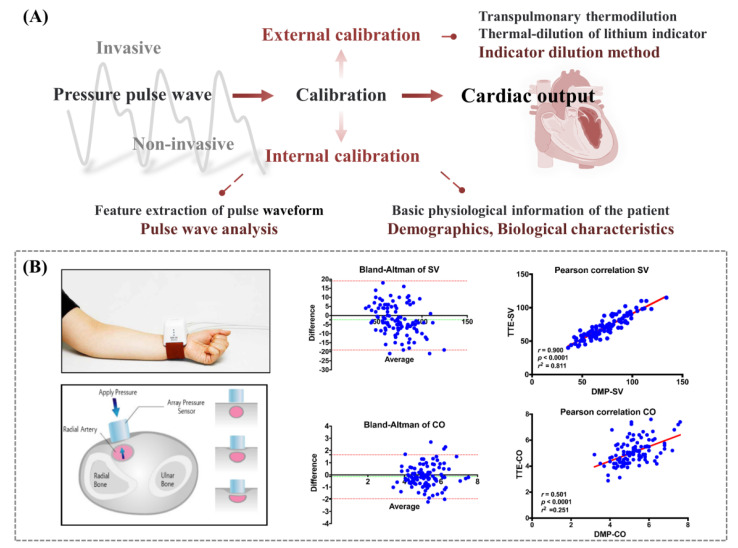
(**A**) Schematic illustration of the calibration methods for cardiac output monitoring. (**B**) Demonstration of the DMP−Life system and Bland–Altman and Pearson’s correlation plots. Reprinted/adapted with permission from Ref. [78].

In contrast, pulse wave analysis allows continuous real-time monitoring of changes in CO. In addition, it can be used for goal-directed haemodynamic therapy in the perioperative period. The pulse wave-based monitoring methods are all based on a common premise that there is a proportional and predictable relationship between the pressure wave and the measurand [77]. As the pressure wave is the result of the interaction between the blood ejected from the heart and the arterial system, its wave characteristics, the harmonic information, are closely related to the characteristic parameter changes in the cardiovascular system. The volume of blood ejected from the left ventricle during a cardiac cycle is known as Stroke Volume (SV), and the product of SV and heart rate (HR) is CO. When using a pulse wave-based monitoring method, it is often necessary to determine the current state of the arterial system by calibrating to correct the pressure-volume relationship for a more accurate estimate of SV (Figure 7). The DMP-Life system (DAEYOMEDI Co., Ansan-si, South Korea) implements continuous arterial blood pressure waveform recording by radial artery tonometry with piezoresistive sensor arrays [78]. CO was estimated using an algorithm that analyzed the systolic part of the arterial blood pressure waveform, taking into account biometric and demographic data. A total of 107 preoperative cardiothoracic surgery patients were enrolled in the study, and the hemodynamic parameters from the radial artery were measured randomly. The Bland–Altman test and Pearson correlation were applied. The results demonstrated that the used blood pressure pulse analysis method is reasonably accurate and consistent with the ultrasound Doppler method in measuring SV and CO.

Since the pulse wave analysis is highly dependent on the signal quality of the arterial blood pressure waveform, active or passive limb movements of the patient may disturb and impair the signal quality, making pulse wave analysis unreliable [76]. With the rapid advances in pulse wave and blood pressure monitoring technology, the development of continuous CO monitoring based on these mechanical sensors remains an attractive option for cardiovascular monitoring in clinical surgery and critical care patients.

## 4. Conclusions and Perspectives

In summary, mechanical sensors have shown promising applications in cardiovascular health monitoring, mainly including pulse wave, blood pressure, heart rhythm, and endocardial pressure. Generally, wearable sensors can be directly attached to the skin surface or integrated with clothing, which is more convenient and has suitable patient compliance [22,79]. By contrast, implantable sensors require surgical trauma but have irreplaceable advantages for tiny force sensing in deep tissues. For the wearable and implantable pressure devices used for diagnostics, measurement accuracy and long-term durability are the main issues hindering their clinical application [80]. In addition, the biocompatibility and flexibility of the sensors need to be further improved to achieve conformal contact with soft tissue/organ surfaces [19,81]. Hence, the application of biomechanical sensors in CVD monitoring still faces great challenges and broad space, and there are optimization trends in the following directions:

(i) Integration of manufacturing miniaturization, wireless transmission to achieve wearable or minimally invasive implantation of sensors, continuous monitoring of multiple physiological indicators; (ii) Incorporation of energy harvesting and conversion strategies to achieve self-powered or low-energy sensing, reducing the charging frequency of implantable sensors; (iii) Extraction of more comprehensive information from multimodal signals, e.g., microvascular flow, to further enrich the functionality of existing sensors, and improve diagnostic accuracy; (iv) During the development of novel biosensors, comparing them with the gold standard methods and conducting the true agreement analysis (Bland–Altman plots) plays an important role in ensuring its accuracy and reliability for medical applications; (v) Development of dynamic monitoring systems using the latest advances in machine learning and cloud computing combined with actual physiological information to respond to the healthcare needs of personalized medicine and remote monitoring. With the contributions of worldwide researchers, wearable and implantable sensors show great prospects for continuous monitoring of cardiovascular disease.
